# Risk of Anorectal Cancer Associated with Benign Anal Inflammatory Diseases: A Retrospective Matched Cohort Study

**DOI:** 10.3390/ijerph19127467

**Published:** 2022-06-17

**Authors:** Wonjeong Chae, Seung Yeon Kang, Sung-In Jang, Yoon Dae Han

**Affiliations:** 1 Department of Health Policy and Management, Graduate School of Public Health, Yonsei University, Seoul 03722, Korea; wjchae0816@yuhs.ac; 2 Institute of Health Services Research, Yonsei University, Seoul 03722, Korea; 3 Division of Colorectal Surgery, Department of Surgery, Severance Hospital, College of Medicine, Yonsei University, Seoul 03722, Korea; sykang120@yuhs.ac; 4 Department of Preventive Medicine, College of Medicine, Yonsei University, Seoul 03722, Korea

**Keywords:** anorectal cancer, benign anal inflammatory diseases, anal fistula, anal fissure

## Abstract

Purpose: The purpose of our study was to evaluate the relationship between benign anal inflammatory diseases and anorectal cancer and assess its risk factors. Methods: A retrospective matched cohort study was conducted that included data from 2002 to 2013. The National Health Insurance Service National Sample Cohort data from 2002 to 2013 was used for the study. Of a total study population of 143,884 individuals, 28,110 individuals with anal fissures were assigned to the case group, while 115,774 individuals without anal fissures were assigned to the control group based on the 1:4 propensity score matching age, sex, and year (case: diagnosed year, control: health service received year). Results: The risk of anorectal cancer was higher in the case group (hazard ratio [HR]: 1.95, 95% confidence interval [CI]: 1.51–2.53) compared to the control group. After grouping anorectal cancers into anal cancer and rectal cancer, the risk remained higher in the case group (anal cancer HR: 2.79, 95% CI: 1.48–5.27; rectal cancer HR: 1.82, 95% CI; 1.37–2.42). The case group was further categorized into patients with fissures and patients with fistulas; patients with fissures showed a higher risk of developing anorectal cancer than patients with fistulas (HR: 2.05, 95% CI: 1.53–2.73 vs. HR: 1.73, 95% CI: 1.13–2.66). Study participants in their 30s and 40s had a 4.19- and 7.39-times higher risk of anorectal cancer compared to those in the higher age groups (0.64–1.84), while patients who did not have inflammatory bowel disease (IBD) had a higher risk of developing anorectal cancer (HR: 2.09, 95% CI: 1.56–2.80). Conclusions and Relevance: Patients with anal fistulas or fissures have an increased risk of being diagnosed with anorectal cancer, especially at a young age and even without IBD.

## 1. Introduction

The American Cancer Society estimated 9090 new cases and 1430 deaths due to anal cancer in 2021 [1]. In Korea, anal cancer is relatively rare accounting for only 0.1% of cancer incidence; however, the incidence of anal cancer has been increasing annually from 354 cases in 1993–1995 to 1062 cases in 2011–2015. Furthermore, the survival rate for anal cancer is poor compared to the survival rate for colorectal cancer. [2,3].

Benign anal lesions, including inflammatory and non-inflammatory diseases, are common perianal diseases that are routinely encountered in clinical practice. In the United Kingdom, the standardized prevalence of anal fistula was 1.80 per 10,000 patients in 2017 [4], and the prevalence of hemorrhoids (another benign anal disease) diagnosed by physicians in Korea was 7.2% [5].

For many years, the possible relationship between benign anal lesions with anal cancer has been debated [6]. However, studies that have evaluated the relationship between benign anal lesions and anal cancers, or other cancers, are limited because of the low prevalence of anal cancer. The majority of previous studies are case control studies that are limited by small study populations and short follow-up times [7,8,9].

Recent results from a Swedish cohort study and a Danish cohort study showed an increased risk of anal cancer for both male and female patients hospitalized for inflammatory benign anal lesions, including anal fissures, fistulas, and perianal abscesses [10,11]. However, hemorrhoids were not associated with an increased risk for anal cancer in a Taiwanese cohort study [12]. Even chronic inflammation and constant irritation were associated with carcinogenesis in general [13]. As per rectal cancer, a previous study discovered that there was an increased risk with borderline significance [12]. In addition, the studies conducted by Danish and Swedish reported that there was no significant association [6,10]. Given the contradictory results from these studies and the rarity of large population-based studies investigating progression from precursor anal lesions to anal cancer in the Asian population, we decided to use data from a large nationwide Korean cohort with a relatively long follow-up period for our analysis.

The purpose of our study was to evaluate the relationship between benign anal inflammatory diseases and anorectal cancer, and to determine the various risk factors for anorectal cancer such as sex, age, region, income level, employment, disability, Charlson comorbidity index (CCI), and inflammatory bowel disease (IBD).

## 2. Materials and Methods

### 2.1. Data Source

The study used the National Health Insurance Service National Sample Cohort (NHIS-NSC) data which includes data on health insurance claims. All Korean citizens are insured under the universal national health insurance service (NHIS). The NHIS-NSC selected 2.2% of the Korean population via systematic stratified random sampling from 1 January 2002 to 31 December 2013. As it is based on the health insurance claim data, it includes diagnostic data, procedure and treatment data, and prescription data. All diagnoses are indicated using the International Statistical Classification of Diseases and Related Health Problems, 10th revision (ICD-10).

### 2.2. Study Population

A total of 143,884 individuals were selected for the final study population. A 2-year wash-out period was applied, and the study population was followed from 2004 onward, regarding the last follow-up date, which could be the cancer diagnosis date for the case group and 31 December 2013, or the death date for the control group. After a 2-year wash-out period, the study included patients with newly diagnosed anal fissures and anorectal cancer cases for the investigation after reducing potential bias.

The case group consisted of 28,110 patients with an anal fissure, and the control group consisted of 115,774 patients without anal fissure. Control group patients were selected based on the 1:4 propensity score matching (PSM) of age, sex, and year (case: diagnosed year, control: health service received year). The matched individuals were assigned to the same index date from the case group that they have the same follow-up start date.

### 2.3. Variables

To define anal fissure and anorectal cancer, ICD-10 K60 and C20 (rectal cancer), and C21 (anus and anal cancer) were used. The anal fissure category was further divided into a fissure (K60.0, K60.1, and K60.2) and fistula (K60.3, K60.4, and K60.5). When an individual had the above diagnostic codes, they were grouped into the respective categories.

Variables such as sex, age, region, income level, employment, disability, CCI, IBD (K64), and cohort entry year were included to adjust the model. The age group was categorized into 10-year intervals from the 20s to the 70s and above. The regions were divided into the capital area (Seoul), metropolitan area (Busan, Daegu, Daejeon, Gwangju, Incheon, and Ulsan), and rural area (Gyeonggi, Gangwon, Chungcheongbuk, Chungcheongnam, Jeollabuk, Jeollanam, Gyeongsangbuk, Gyeongsangnam, and Jeju). CCI were divided into two groups based on 3.

### 2.4. Statistical Analysis

**Figure 1:**
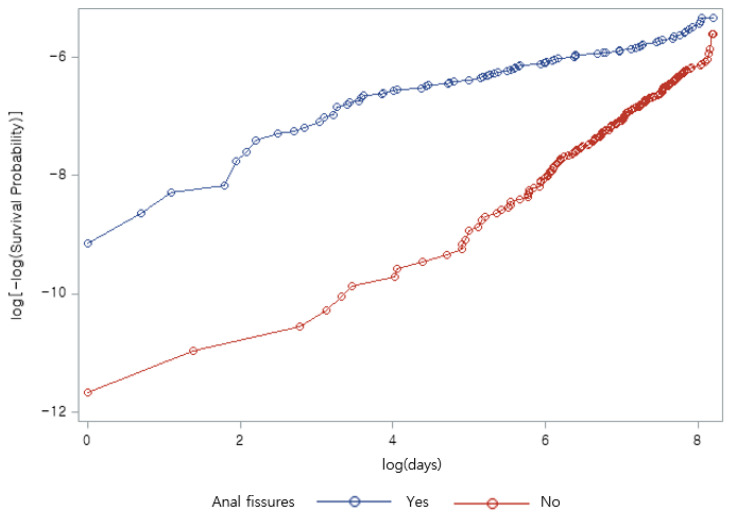
Log-log survival probability test for anal fissures.

Descriptive statistics and chi-square tests were applied to test differences in variables and incidents of anorectal cancer at the baseline. The investigation of the association between anal fissure and anorectal cancer considering the survival time was performed with Kaplan-Meier curves and a log-rank test. The multivariate Cox proportional hazard model was employed to estimate adjusted hazard ratios (HRs) and 95% confidential intervals (CI), including all covariates. To test the proportional hazard assumption, we performed a log-log survival probability test and the result showed it was not validated (Figure 1). All analyses were performed using SAS software, version 9.4 (SAS Institute, Cary, NC, USA).

### 2.5. Ethical Statement

The data used for the study is distributed by NHIS, which is available upon request for research purposes. The format of data is de-identified secondary health insurance claim data. Thus, the study did not require approval by the Institutional Review Board of the organization (IRB number: 4-2021-1414).

## 3. Results

### 3.1. Patient Characteristics

Table 1 presents the general characteristics of the study population. A total of 143,884 individuals were included in the study; the case group constituted 19.5% of the study population (*n* = 28,110) and the control group constituted the remaining 80.5% (115,774). Among the case group, 73.4% (*n* = 20,627) and 26.6% (*n* = 7483) were patients with fissures and fistulas, respectively. Of the total study population, 0.2% (*n* = 250) were diagnosed with anorectal cancer, where anorectal cancer was identified in 0.3% (*n* = 92) of the case group and 0.1% (*n* = 158) of the control group. Individuals diagnosed with IBD constituted 1.5% (*n* = 2125) of the total study population, of which 0.5% (*n* = 11) had anorectal cancer. Of those who did not have IBD, out of 141,795 individuals, 0.2% (*n* = 239) had anorectal cancer. General population of matched population is shown on Supplementary Table S1.

### 3.2. Anal Fissure Associated with Anorectal Cancer Development

**Figure 2:**
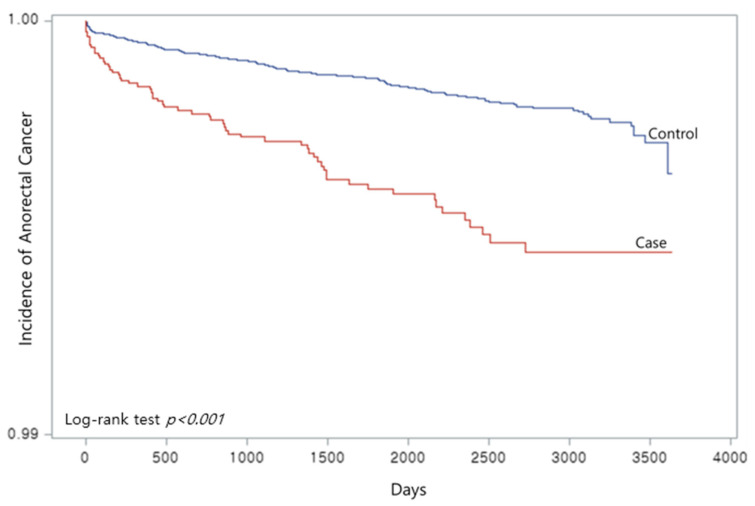
Kaplan-Meier curve on anal fissure and anorectal cancer.

The Kaplan-Meier curves (red: case; blue: control) showed that the case group was at a higher risk of developing anorectal cancer (Figure 2). The log-rank test result showed a *p*-value below 0.0001.

The analyses were conducted to examine the risk of developing anorectal cancer and anal cancer and rectal cancer specifically. Table 2 exhibited the results of the Cox proportional hazard model presented with HRs. The risk of developing anorectal cancer was higher in the case group (HR: 1.95, 95% CI: 1.51–2.53). As for anal cancer and rectal cancer, the case group had a higher risk compared to the control group (anal cancer HR: 2.79, 95% CI: 1.48–5.27; rectal cancer HR: 1.82, 95% CI: 1.37–2.42).

In subdivisions of anal fissure, the risk of anorectal cancer was at HR 2.05 (95% CI: 1.53–2.73) and 1.73 (95% CI: 1.13–2.66) for fissure and fistula accordingly. Moreover, the risk of anal cancer was higher in patients with fistulas (HR: 4.06, 95% CI: 1.77–9.30; fissure, HR: 2.23, 95% CI: 1.04–4.77). As for rectal cancer, the risk was higher in fissure patients (HR: 2.01, 95% CI: 1.47–2.75; fistula, HR: 1.37, 95% CI: 0.83–2.28).

Regarding age and IBD, individuals who were above 50 years old had a higher risk of developing anorectal cancer (50s, HR: 1.65, 95% CI: 1.16–2.35; 60s, HR: 2.29, 95% CI: 1.55–3.39; 70s and above, HR: 3.99 95% CI: 2.61–6.11). Also, patients with IBD had a higher risk of developing anorectal, yet this did not result in a statistically significant outcome (HR: 1.78, 95% CI: 0.97–0.3.26).

### 3.3. Subgroup Analyses on Anorectal Cancer Associated with Anal Fissure

Sub-group analyses on the association between anal fissure and different types of cancer were conducted (Table 3). Results expressed that the risk of cancers was higher in female patients compared to male patients (anorectal cancer, HR: 2.52, 95% CI: 1.63–3.89; anal cancer, HR: 3.58, 95% CI: 1.18–10.78; rectal cancer, HR: 2.35, 95% CI: 1.46–3.77). Regarding age group, patients who were in their 40s, 50s, and above 70 years had a higher risk of developing anorectal cancer (40s, HR: 2.30, 95% CI: 1.34–3.94; 50s, HR: 2.13, 95% CI: 1.31–2.45; above 70 years, HR: 2.40, 95% CI: 1.30–4.46). Moreover, patients who were in their 40s showed the highest risk for anal cancer (HR: 7.39, 95% CI: 1.86–29.33), and patients who were in their 50s and above 70 years showed higher risks (50s, HR: 2.20, 95% CI: 1.28–3.78; above 70 years, HR: 2.37, 95% CI: 1.26–4.47) for rectal cancer.

Table 4 presents another subgroup analysis on the risk of anorectal cancer divided on the basis of fissure and fistula. As for fissure, the risk was higher in female patients (male, HR: 1.68, 95% CI: 1.14–2.48; female, HR: 2.67, 95% CI: 1.71–4.16). Patients whose CCI was above 3 had a higher risk for anorectal cancer (HR: 2.11, 95% CI: 1.56–2.80), while patients who did not have IBD had a higher risk of developing anorectal cancer (HR: 2.09, 95% CI: 1.56–2.80). Regarding the presence of fistula, male patients showed a higher risk of developing anorectal cancer (HR: 1.69, 95% CI: 1.07–2.68). The trend of anorectal cancer associated with CCI and IBD among patients with fistulas was similar to that observed in patients with fissures (CCI above 3, HR: 1.63, 95% CI: 1.63, 95% CI: 1.03–2.55; no IBD, HR: 1.76, 95% CI: 1.14–2.72).

## 4. Discussion

Anal fistulas and fissures are common types of benign anal lesions that are typically infected and commonly cause chronic inflammation. In our nationwide retrospective matched cohort study, both anal fistula and fissure elevated the risk of anorectal cancer. Due to the low incidence of anorectal cancer itself, there are not many studies on the association between benign inflammatory anorectal disease and anorectal cancer. Several case reports discuss cases of chronic benign inflammatory anorectal disease (especially non-healing types or in an atypical position with induration or ulceration) that are ultimately diagnosed as anorectal cancer [14,15]. However, it is difficult to know whether the cancer was previously misdiagnosed, or developed as a result of ongoing chronic inflammation.

There is growing evidence to support the increased cancer risk of chronic benign inflammation [16,17,18]. Chronic inflammation and constant irritation are hypothesized to be associated with anorectal cancer [13]. In chronic inflammation, macrophages, lymphocytes, and plasma cells infiltrate the damaged tissue, and persistent pathological activation of macrophage may result in continuous tissue damage [19]. Subsequently, macrophages release nitric oxide, which directly damages DNA and can lead to the inhibition of apoptosis and may stimulate angiogenesis [20]. Moreover, with the generation of inflammatory cytokines, prostaglandins, and reactive oxygen species, a repressed immune response can promote tumor growth [21].

This study suggests that the overall rate of anorectal cancer was higher in women than men. Women with benign inflammatory anorectal disease were more exposed to the risk of anorectal cancer with a HR of 2.53. Developing cancer may also be associated with human papillomavirus (HPV) infection [22]. Anal squamous intraepithelial lesions are HPV associated lesions that can be further classified as anal intraepithelial neoplasia (AIN), which is a precursor of squamous cell carcinoma (SCC) of the anus [23]. Benign inflammatory anorectal disease may facilitate viral access to the epithelium and potentially act as a cofactor to promote HPV-related carcinogenesis or interplay with inflammation [11]. This additional risk factor for women may have increased the risk of anorectal cancer. Even when excluding common risk factors such as anal sex, genital warts, and cigarette smoking, the anatomic proximity of the vaginal introitus to the anus may be related to the non-sexual and autoinoculation of the virus in women via vaginal secretion, and this may be hypothesized as the reason for women being at a higher risk of anorectal cancer than men [24]. A study from Scotland also showed that anal SCC doubled to 0.37 per 100,000 in men and 0.55 in women [25].

Regarding age group, the overall anorectal cancer risk was highest in the 40s age group. For anal cancer specifically, the risk of anorectal cancer in individuals belonging to the 30s and 40s age groups was 4.19- and 7.39-times higher risk compared to those in the 50s, 60s, and 70s age groups (0.64–1.84). Although only few data are available, a meta-analysis reported that anal intercourse in all sex acts in a month varies from 1.1–20.6%. [26] Nevertheless, Korea is considered to have a low rate of homosexual intercourse compared to western countries. HPV increases the incidence of anal cancer in all individuals, despite sexuality. Since HPV is primarily a sexually transmitted disease, it may be assumed that frequent sexual intercourse increases the transmission of HPV, and thus, the risk of anorectal cancer [27,28]. Additionally, a recent study reported the frequency of sexual intercourse per month in Korea. On average, individuals in the 50 s age group had sexual intercourse less than 3 times per month; however, the frequency of sexual intercourse was highest for individuals in the 30s and 40s age groups [29,30], which may be a plausible explanation for the increased risk of anorectal cancer observed in our study for these age groups.

From previous studies, IBD is known to be associated with colorectal cancer [31,32]; however, not many studies have examined the association of IBD with anorectal cancer. One study suggests that for patients diagnosed with anal and/or perianal Crohn’s disease, the risk of anorectal cancer was 11 times greater than the risk of colon cancer [33]. In our study, anorectal cancer occurred in 239 patients (0.2%) out of 141,795 patients without IBD. Considering 11 patients with anorectal cancer also had IBD (0.5%), there is a higher probability of developing cancer for patients who have an anal fissure and/or fistula without IBD.

Our study highlights the risk of anorectal cancer for anal fistula or fissure in young individuals in their 30s and 40s, without IBD. However, our study has several limitations. The data used for the study is health insurance claim data which is provided by the Korean government. Therefore, the sensitive information or health behavior related data were not given. Such as the pathology of anorectal cancer was not reported. In addition, Korea has a low incidence of human immunodeficiency virus (HIV) infection with low and undetected men who have sex with men [30]. The status of HIV infection (positive or negative) was also difficult to reflect on results. Even though anal cancer is one of the rare conditions, a regular medical checkup should be considered.

## Figures and Tables

**Table 1:** General characteristics of the study population.

KERRYPNX		Total (*n*, %)		Anorectal Cancer				
				Yes	%	No	%	*p*-Value
		143,884		250	0.2	143,634	99.8	
Anal Inflammatory Disease								<0.0001
	Yes	28,110	19.5	92	0.3	28,018	99.7	
	Fissure	20,627	73.4	67	0.3	20,560	99.7	0.9042
	Fistula	7483	26.6	25	0.3	7458	99.7	
	No	115,774	80.5	158	0.1	115,616	99.9	
Sex								<0.0001
	Male	75,941	52.8	166	0.2	75,775	99.8	
	Female	67,943	47.2	84	0.1	67,859	99.9	
Age group								<0.0001
	20s	38,229	26.6	8	0.0	38,221	100.0	
	30s	40,420	28.1	22	0.1	40,398	99.9	
	40s	32,393	22.5	56	0.2	32,337	99.8	
	50s	19,930	13.9	70	0.4	19,860	99.6	
	60s	8633	6.0	52	0.6	8581	99.4	
	70s~	4279	3.0	42	1.0	4237	99.0	
Income level								0.3194
	Low	22,097	15.4	33	0.1	22,064	99.9	
	Mid	68,186	47.4	113	0.2	68,073	99.8	
	High	53,601	37.3	104	0.2	53,497	99.8	
Employment								0.7919
	Yes	80,040	55.6	137	0.2	79,903	99.8	
	No	63,844	44.4	113	0.2	63,731	99.8	
Regions								0.5346
	Capital	65,254	45.4	116	0.2	65,138	99.8	
	Urban	37,102	25.8	57	0.2	37,045	99.8	
	Rural	41,528	28.9	77	0.2	41,451	99.8	
Disabled								<0.0001
	Yes	6331	4.4	25	0.4	6306	99.6	
	No	137,553	95.6	225	0.2	137,328	99.8	
CCI †								<0.0001
	less than 3	89,096	61.9	16	0.0	89,080	100.0	
	3 or more	54,788	38.1	234	0.4	54,554	99.6	
IBD ‡								0.0001
	Yes	2125	1.5	11	0.5	2114	99.5	
	No	141,759	98.5	239	0.2	141,520	99.8	
Cohort entry year								<0.0001
	2004	14,408	10.0	43	0.3	14,365	99.7	
	2005	14,054	9.8	35	0.2	14,019	99.8	
	2006	13,552	9.4	28	0.2	13,524	99.8	
	2007	13,440	9.3	31	0.2	13,409	99.8	
	2008	13,641	9.5	28	0.2	13,613	99.8	
	2009	15,085	10.5	20	0.1	15,065	99.9	
	2010	14,844	10.3	24	0.2	14,820	99.8	
	2011	15,318	10.6	22	0.1	15,296	99.9	
	2012	15,268	10.6	11	0.1	15,257	99.9	
	2013	14,274	9.9	8	0.1	14,266	99.9	

† Charlson comorbidity index; ‡ Inflammatory bowel disease.

**Table 2:** Result of Cox Hazzard Regression on Anorectal Cancer and sub-cancers.

		Anorectal Cancer		Anal Cancer		Rector Cancer	
		HR	95% CI	HR	95% CI	HR	95% CI
Anal Inflammatory Disease							
	Yes	1.95	(1.51–2.53)	2.79	(1.48–5.27)	1.82	(1.37–2.42)
	Fissure	2.05	(1.53–2.73)	2.23	(1.04–4.77)	2.01	(1.47–2.75)
	Fistula	1.73	(1.13–2.66)	4.06	(1.77–9.30)	1.37	(0.83–2.28)
	No	1.00	-	1.00	-	1.00	-
Sex							
	Male	1.57	(1.17–2.10)	2.10	(1.00–4.43)	1.49	(1.08–2.04)
	Female	1.00	-	1.00	-	1.00	-
Age group							
	20s	0.21	(0.10–0.44)	0.13	(0.02–1.01)	0.23	(0.10–0.51)
	30s	0.40	(0.24–0.66)	0.49	(0.17–1.45)	0.38	(0.22–0.66)
	40s	1.00	-	1.00	-	1.00	-
	50s	1.65	(1.16–2.35)	1.67	(0.73–3.81)	1.63	(1.10–2.41)
	60s	2.29	(1.55–3.39)	1.26	(0.46–3.48)	2.54	(1.66–3.89)
	70s~	3.99	(2.61–6.11)	0.77	(0.16–3.70)	4.89	(3.12–7.67)
Income level							
	Low	1.00	-	1.00	-	1.00	-
	Mid	1.29	(0.87–1.92)	1.73	(0.59–5.08)	1.24	(0.81–1.89)
	High	1.10	(0.74–1.64)	1.15	(0.37–3.53)	1.12	(0.73–1.72)
Employment							
	Yes	1.00	(0.75–1.33)	0.51	(0.25–1.04)	1.13	(0.83–1.55)
	No	1.00	-	1.00	-	1.00	-
Regions							
	Capital	1.00	-	1.00	-	1.00	-
	Urban	0.85	(0.62–1.17)	0.36	(0.14–0.94)	0.98	(0.70–1.38)
	Rural	0.89	(0.67–1.19)	0.56	(0.27–1.18)	0.98	(0.71–1.34)
Disabled							
	Yes	1.07	(0.70–1.63)	2.62	(1.12–6.13)	0.86	(0.53–1.41)
	No	1.00	-	1.00	-	1.00	-
CCI †							
	less than 3	1.00	-	1.00	-	1.00	-
	3 or more	12.40	(7.39–20.80)	10.55	(3.16–35.21)	12.79	(7.21–22.68)
IBD ‡							
	Yes	1.78	(0.97–3.26)	3.07	(0.94–10.03)	1.53	(0.75–3.10)
	No	1.00	-	1.00	-	1.00	-
Cohort Entry Year							
	2004	1.00	-	1.00	-	1.00	-
	2005	0.93	(0.59–1.48)	0.99	(0.32–3.07)	0.93	(0.56–1.54)
	2006	0.82	(0.50–1.34)	0.70	(0.20–2.50)	0.84	(0.49–1.44)
	2007	0.98	(0.60–1.60)	1.12	(0.35–3.53)	0.96	(0.56–1.64)
	2008	1.00	(0.60–1.67)	0.94	(0.28–3.17)	1.02	(0.58–1.79)
	2009	0.81	(0.46–1.42)	0.43	(0.09–2.18)	0.90	(0.49–1.65)
	2010	1.08	(0.63–1.85)	0.75	(0.18–3.13)	1.15	(0.64–2.07)
	2011	1.21	(0.69–2.11)	1.22	(0.33–4.59)	1.21	(0.65–2.25)
	2012	0.77	(0.38–1.55)	n/a	-	0.96	(0.46–1.99)
	2013	1.11	(0.50–2.47)	0.58	(0.07–5.12)	1.28	(0.54–3.03)

† Charlson comorbidity index; ‡ Inflammatory bowel disease.

**Table 3:** Subgroup analyses on anorectal caner, anal cancer, and rector cancer.

		Anorectal Cancer		Anal Cancer		Rector Cancer	
		HR	95% CI	HR	95% CI	HR	95% CI
Sex							
	Male	1.69	(1.22–2.33)	2.43	(1.11–5.31)	1.57	(1.10–2.24)
	Female	2.52	(1.63–3.89)	3.58	(1.18–10.78)	2.35	(1.46–3.77)
Age group							
	20s	0.93	(0.18–4.67)	n/a	-	0.40	(0.05–3.35)
	30s	1.34	(0.69–4.00)	4.19	(0.68–25.64)	1.26	(0.44–3.62)
	40s	2.30	(1.34–3.94)	7.39	(1.86–29.33)	1.78	(0.97–3.29)
	50s	2.13	(1.31–2.45)	1.84	(0.61–5.52)	2.20	(1.28–3.78)
	60s	1.47	(0.80–2.69)	0.64	(0.07–5.21)	1.64	(0.87–3.10)
	70s~	2.40	(1.30–4.46)	1.41	(0.09–23.57)	2.37	(1.26–4.47)
CCI †							
	less than 3	1.51	(0.48–4.79)	2.86	(0.26–31.82)	1.33	(0.36–4.96)
	3 or more	1.97	(1.51–2.56)	2.80	(1.45–5.41)	1.84	(1.37–2.46)
IBD ‡							
	Yes	1.17	(0.34–4.05)	3.66	(0.31–42.86)	0.71	(0.14–3.64)
	No	1.99	(1.53–2.59)	2.73	(1.41–5.29)	1.88	(1.40–2.50)

† Charlson comorbidity index; ‡ Inflammatory bowel disease.

**Table 4:** Subgroup analyses on anorectal cancer by anal fissure and fistula.

		Fissure		Fistula	
		HR	95% CI	HR	95% CI
Sex					
	Male	1.68	(1.14–2.48)	1.69	(1.07–2.68)
	Female	2.67	(1.71–4.16)	1.56	(0.48–5.02)
Age group					
	20s	1.21	(0.24–6.15)	n/a	-
	30s	1.73	(0.66–4.55)	1.51	(0.34–6.79)
	40s	2.39	(1.31–4.39)	2.12	(0.93–4.82)
	50s	2.26	(1.31–3.91)	1.86	(0.87–3.98)
	60s	1.60	(0.81–3.16)	1.20	(0.42–3.40)
	70s~	2.38	(1.21–4.67)	2.48	(0.85–7.23)
CCI †					
	less than 3	0.57	(0.07–4.50)	3.20	(0.88–11.68)
	3 or more	2.11	(1.58–2.83)	1.63	(1.03–2.55)
IBD ‡					
	Yes	1.16	(0.30–4.56)	1.21	(0.14–10.26)
	No	2.09	(1.56–2.80)	1.76	(1.14–2.72)

† Charlson comorbidity index; ‡ Inflammatory bowel disease.

## Data Availability

Data used for this study is available upon request to NHIS (https://nhiss.nhis.or.kr, accessed on 4 October 2021) for research purpose only.

## Supplementary Materials

The following supporting information can be downloaded at: https://www.mdpi.com/article/10.3390/ijerph19127467/s1, Table S1. General population of matched population.
