# Supplementary material for: Risk of Anorectal Cancer Associated with Benign Anal Inflammatory Diseases: A Retrospective Matched Cohort Study
Source: Int J Environ Res Public Health. 2022 Jun 17;19(12):7467. doi: 10.3390/ijerph19127467 (PMC9223752; doi:10.3390/ijerph19127467)
Supplement: Supplementary file 1 [file ijerph-19-07467-s001.zip › ijerph-1726041-supplementary.pdf]

Supplementary material

Table S1. General population of matched population

	Anal fissures						p-value
	Total (N)		Yes (n, %)		No (n,%)		
	143884		28110	19.5	115774	80.5	
Sex							0.9604
Male	75941	52.8	14840	19.5	61101	80.5	
Female	67943	47.2	13270	19.5	54673	80.5	
Age group							0.8359
20s	38229	26.6	7428	19.4	30801	80.6	
30s	40420	28.1	7848	19.4	32572	80.6	
40s	32393	22.5	6346	19.6	26047	80.4	
50s	19930	13.9	3918	19.7	16012	80.3	
60s	8633	6.0	1712	19.8	6921	80.2	
70s ~	4279	3.0	858	20.1	3421	79.9	
Income level							<.0001
Low	22097	15.4	3773	17.1	18324	82.9	
Mid	68186	47.4	12974	19.0	55212	81.0	
High	53601	37.3	11363	21.2	42238	78.8	
Employment							<.0001
Yes	80040	55.6	16170	20.2	63870	79.8	
No	63844	44.4	11940	18.7	51904	81.3	
Regions							<.0001
Capital	65254	45.4	13529	20.7	51725	79.3	
Urban	37102	25.8	6678	18.0	30424	82.0	
Rural	41528	28.9	7903	19.0	33625	81.0	
Disabled							0.0216
Yes	6331	4.4	1166	18.4	5165	81.6	
No	137553	95.6	26944	19.6	110609	80.4	
CCI†							<.0001
less than 3	89096	61.9	14859	16.7	74237	83.3	
3 or more	54788	38.1	13251	24.2	41537	75.8	
IBD‡							<.0001
Yes	2125	1.5	695	32.7	1430	67.3	
No	141759	98.5	27415	19.3	114344	80.7	
Cohort Entry Year							0.7698
2004	14408	10.0	2810	19.5	11598	80.5	
2005	14054	9.8	2752	19.6	11302	80.4	
2006	13552	9.4	2636	19.5	10916	80.5	
2007	13440	9.3	2621	19.5	10819	80.5	
2008	13641	9.5	2634	19.3	11007	80.7	
2009	15085	10.5	2932	19.4	12153	80.6	
2010	14844	10.3	2881	19.4	11963	80.6	
2011	15318	10.6	2986	19.5	12332	80.5	
2012	15268	10.6	2966	19.4	12302	80.6	
2013	14274	9.9	2892	20.3	11382	79.7	

†Charlson comorbidity index; ‡Inflammatory bowel disease.
